# Latin American interventions in children and adolescents’ sedentary behavior: a systematic review

**DOI:** 10.11606/s1518-8787.2020054001977

**Published:** 2020-05-28

**Authors:** Evelyn Helena Corgosinho Ribeiro, Paulo Henrique Guerra, Ana Carolina de Oliveira, Kelly Samara da Silva, Priscila Santos, Rute Santos, Anthony Okely, Alex Antonio Florindo

**Affiliations:** I Universidade de São Paulo Grupo de Estudos e Pesquisas Epidemiológicas em Atividade Física e Saúde São PauloSP Brasil Universidade de São Paulo. Grupo de Estudos e Pesquisas Epidemiológicas em Atividade Física e Saúde. São Paulo, SP, Brasil; II Universidade Federal da Fronteira Sul ChapecóSC Brasil Universidade Federal da Fronteira Sul. Chapecó, SC, Brasil; III Escola de Artes, Ciências e Humanidades Universidade de São Paulo São PauloSP Brasil Escola de Artes, Ciências e Humanidades da Universidade de São Paulo. São Paulo, SP, Brasil; IV Universidade Federal de Santa Catarina Núcleo de Pesquisa em Atividade Física e Saúde FlorianópolisSC Brasil Universidade Federal de Santa Catarina. Núcleo de Pesquisa em Atividade Física e Saúde. Florianópolis, SC, Brasil; V Universidade do Porto Centro de Investigação em Actividade Física, Saúde e Lazer Porto Portugal Universidade do Porto. Centro de Investigação em Actividade Física, Saúde e Lazer. Porto, Portugal; VI Universidade Lusófona Lisboa Portugal Universidade Lusófona. Lisboa, Portugal; VII University of Wollongong Wollongong Australia University of Wollongong. Early Start. Wollongong, Australia

**Keywords:** Child, Adolescent, Sedentary Behavior, Evaluation of the Efficacy-Effectiveness of Interventions, Systematic Review

## Abstract

**OBJECTIVE:**

To identify and evaluate the effects of community-based interventions on the sedentary behavior (SB) of Latin American children and adolescents.

**METHODS:**

A systematic review on community-based trials to reduce and/or control SB in Latin American countries (Prospero: CRD42017072157). Five databases (PubMed, Web of Science, Scopus, SciELO and Lilacs) and a reference lists were searched.

**RESULTS:**

Ten intervention studies met the eligibility criteria and composed the descriptive synthesis. These studies were conducted in Brazil (n=5), Mexico (n=3), Ecuador (n=1) and Colombia (n=1). Most interventions were implemented in schools (n=8) by educational components, such as meetings, lessons, and seminars, on health-related subjects (n=6). Only two studies adopted specific strategies to reduce/control SB; others focused on increasing physical activity and/or improving diet. Only one study used an accelerometer to measure SB. Seven studies investigated recreational screen time. Eight studies showed statistically significant effects on SB reduction (80%).

**CONCLUSIONS:**

Latin America community-based interventions reduced children and adolescents’ SB. Further studies should: define SB as a primary outcome and implement strategies to reduce such behaviour; focus in different SBs and settings, other than recreational screen time or at-home sitting time; and use objective tools together with questionnaires to measure sedentary behaviour in.

## INTRODUCTION

High levels of sedentary behavior (SB) –activities in a seated or reclining position requiring low energy expenditure^1^ are associated with cardiovascular diseases, diabetes and premature mortality risk^2^. A study that analyzed over one million people reported that high activity level (60 to 75 minutes per day) attenuate, but does not eliminate, the increased mortality risk associated with high TV-viewing time ( ≥ 3 hours per day)^2^. This type of SB is very common among children and adolescents^3^.

SB in childhood and adolescence is related to overweight and obesity, insufficient levels of physical activity (PA), unhealthy food consumption, and poor academic performance and perceptions of well-being^4^. A systematic review reported that SB also plays a role in weight gain from childhood to adulthood^8^.

Among children and adolescents, SB is usually assessed by self-reported recreational screen time (e.g., TV-viewing, using computer, tablet or smartphone for non-school work, or playing electronic games) or objective measurements (e.g., accelerometers as ActiGraph and ActivPAL)^9^, which provide information on total SB time but does not discriminate the type of activity and its context. The contexts in which young people are usually sedentary are little explored, such as sitting time at home, at school, and during transportation^3^.

Guidelines from several countries state that children and adolescents should spend less than two hours a day in recreational screen time^10,11^, as well as limit sedentary transport, sitting time, and indoors time during the day^10^. Yet, studies conducted in high-income countries showed that youth spend from two to four hours a day in recreational screen time and are sedentary from five to ten hours daily^3^.

In Latin America, over 50% of children and adolescents do not follow the recommendation of < 2 hours a day using electronic media for recreational purposes^12^. Higher levels of recreational screen time appear to be more prevalent among girls, adolescents, urban area residents, and less active individuals^13^. Tracking shows that SB increases with age, and that childhood and adolescence lifestyles are maintained during adulthood^13,17^. Such findings indicate that preventive efforts need to commence as soon as possible to educate and support children in maintaining healthy levels of recreational screen time and overall sitting time.

Intervention studies are key to identify effective strategies in reducing high SB levels. Regarding children and adolescents, most interventions are implemented in schools and communities. Systematic reviews have shown the potential of strategies in reducing recreational screen time among children and adolescents, such as classroom sessions, educational newsletter, homework assignments for parents, counseling practices, and TV-viewing time reduction^21^. However, most of these studies were conducted in high-income countries^20,21^, hampering the generalization of their findings into low-, middle- and upper-middle- income countries, as they differ in potential correlates of SB and acquire less available resources to support potential interventions^22^.

Reducing SB is a global goal and Latin America low-, middle- and upper-middle-income countries, as Brazil and Mexico, are testing strategies to achieve it, but the results of these interventions have not yet been summarized. This study aimed to identify and evaluate the effects of community-based interventions to reduce or control SB among children and/or adolescents in Latin American countries.

## METHODS

### Study Design

This systematic literature review followed the Prisma (Preferred Reporting Items for Systematic Reviews and Meta-Analyses) protocol and was registered in Prospero (CRD42017072157).

The following databases were searched: PubMed, Web of Science, Scopus, SciELO and Lilacs. Systematic searches combined keywords for type of study, SB and population: (((intervention[Text Word]) OR strategy[Text Word])) AND ((((((sedentary behavior[Text Word]) OR sitting time[Text Word]) OR screen time[Text Word]) OR television[Text Word]) OR computer[Text Word]) OR videogame[Text Word]). Activated filters: Clinical Trial; Controlled Clinical Trial; Pragmatic Clinical Trial; Randomized Controlled Trial; Humans; Child: birth-18 years. The document detailing all strategies applied can be requested by email to the corresponding author. Searches were filtered and/or performed in English, Portuguese and Spanish. To avoid potential losses, articles that were assessed by its full-texts had their reference lists checked (manual search). Searches in Google Scholar were also performed.

### Selection process and data extraction

Inclusion criteria were: (i) intervention studies (experimental and quasi-experimental); (ii) implemented in community settings (e.g.: school, public clubs/parks, primary health care centers); (iii) in which primary or secondary objective was reducing SB; (iv) conducted with children and adolescents (< 18 years old); (v) in Latin American countries and; (vi) published until May 2019.

Three researchers, organized in two pairs (EHCR–ACMO; EHCR–PCS), assessed titles, abstracts, full texts and data collection. A senior reviewer (PHG) solved doubts and disagreements.

Data were collected in a structured spreadsheet, organized as follows: (i) sample characterization, study site (city/country) and primary object; (ii) study type, number and type of settings (school, church, home), duration and description of the intervention and control group; (iii) SB assessment method and number of individuals included in the analyzes; and (iv) description of SB results. When available, study protocols were consulted.

Two reviewers independently assessed the risk of bias using an adapted version of the Effective Public Health Practice Project (EPHPP) instrument^23,24^. This instrument analyzes important domains of intervention studies (selection, study design, confounders, blinding of assessors, data collection methods, withdrawals and drop-outs, analyses) and ranks the information as low, moderate and high risk of bias. The adjusted EPHPP can be requested by contacting the corresponding author.

## RESULTS

**Figure 1:**
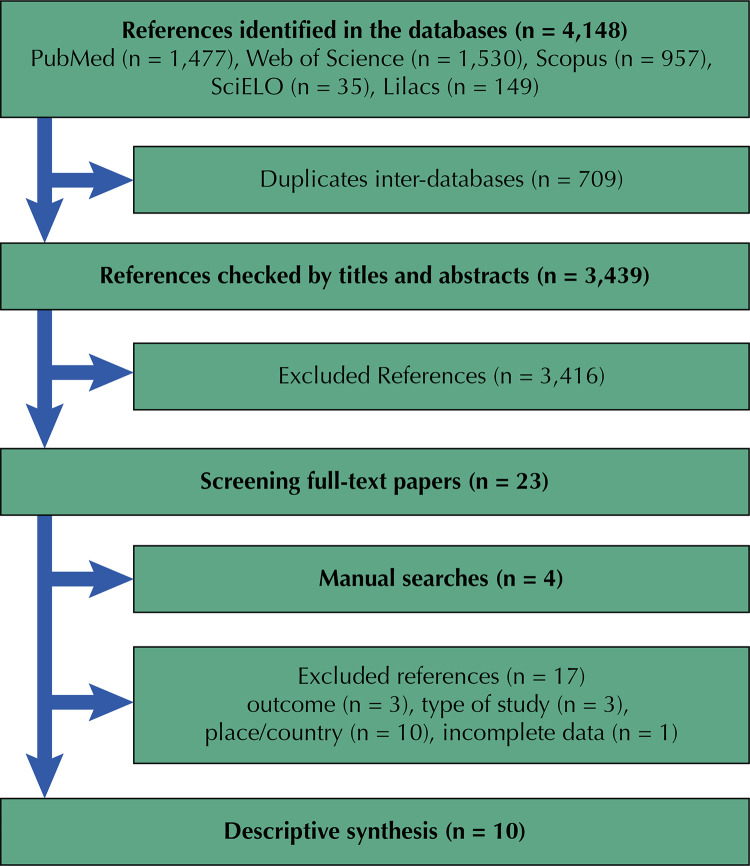
Systematic review flowchart.

Figure 1 shows the flowchart. Of the 4,148 potential references, 709 duplicates were removed and 3,439 selected for title and abstract screening. After screening, 27 studies were referred for full text assessment, of which 17 were removed (reasons: outcome [n=3], study design [n=3], country [n=10], incomplete data [n=1]) and 10 selected for the descriptive syntheses. All included studies were cluster randomized controlled trials.

Interventions were implemented in Brazil (n=5)^25^, Mexico (n=3)^30^, Ecuador (n=1)^33^, and Colombia (n=1)^34^. Most studies involved adolescents and were focused more on girls than boys^25-27,33^. Five studies had SB as primary objective^25,28,32^(Table 1).

**Table 1:** Descriptive characteristics of included studies.

Reference	Program	Location (city/country)	Mean age (years)	% Females in sample	Primary objective of the manuscript
Colín-Ramírez et al.^32^	RESCATE	Mexico City/Mexico	9.4	48	PA and Screen time
Bacardi-Gascon et al.^30^	---	Tijuana/Mexico	8.5	49	BMI, PA and Food Consumption
Hardman et al.^25^	Saúde na Boa	Recife and Florianopolis/Brazil	18.4	56	Screen time
Martínez-Andrade et al.^31^	Creciendo Sanos	Mexico City/Mexico	3.4	47	Obesity, PA and Diet
Andrade et al.^33^	ACTIVITAL	Cuenca/Ecuador	12.8	68	Screen time
Leme et al.^26^	Healthy Habits, Healthy Girls Brazil	Sao Paulo/Brazil	16.1	100	BMI
Guimarães et al.^27^	---	Campinas/Brazil	16.5	75	PA and Cardiovascular Risk Factors
Bandeira et al.^28^	Fortaleça sua Saúde	Fortaleza/Brazil	11-17	49	Screen time
Gutiérrez-Martínez et al.^34^	Estudio Internacional de Obesidad Infantil, Estilos de Vida Y Medio Ambiente (ISCOLE)	Bogota/Colombia	10.5	57.5	PA, SB and Adiposity
Rauber et al.^29^	HEPchild	Federal District/Brazil	9 -11	62.5	PA and Diet

PA=Physical Activity; BMI=Body Mass Index; SB=Sedentary Behavior

### Description of the interventions

Most interventions were school-based (n=8)^25^ and their lengths ranged from five days to 28 months. Five studies lasted at least six months^25,26,30,32,33^. Most studies (n=9) allocated participants into control and intervention groups. Rauber et al. (2018)^29^ allocated into the intervention group participants who answered to advertisements on a regional television channel. Other study applied the same protocol of the intervention group to participants allocated at baseline to the control group, due to the benefits of the intervention^30^. One study measured SB by an accelerometer (GT3x+, ActiGraph)^34^, but the others applied questionnaires. Recreational screen time was the most investigated behavior (television, computer and videogame)^25,26,28,30^. In five studies, over 70% of participants (intervention and control group) completed the intervention^26,28^. Six studies performed their analyses following intention-to-treat principles^25,26,30,31,33,34^.

Educational components (meetings/lessons/seminars on health-related subjects) (n=7)^26^ and parents involvement (n=7)^26,28^ were the most applied strategies, followed by information (posters, newsletters, guidelines) (n=4)^25,26,28,33^ and extra physical education/PA sessions (n=5)^26^(Table 3). In Martinez-Andrade et al. (2014)^31^, the intervention protocol boiled down to workshops with parents to modify their children PA behavior and dietary habits (aged from 2 to 5). The least applied strategies were: sending healthy messages to mobile phones (n=2)^26,34^, providing exercise breaks in the classroom (n=1)^3^, and offering PA/sports events on weekends (n=1)^25^.

**Table 2:** Interventions’ General Characteristics.

	Setting	Population	Intervention (months)	Follow-up (months)	Methods of measurement	Assessed Behaviors	Sample INT/CN	Adherence to protocol (%)	ITT analysis
Colín-Ramírez et al.^32^	School	Children	12	-	Questionnaire	Screen time	245/253	39.6	No
Bacardi-Gascon et al.^30^	School	Children	6	18	Questionnaire	Sitting/Screen time	280/252	91.4	Yes
Hardman et al.^25,35,36^	School	Adolescents	9	-	Questionnaire	Screen time	1059/1096	44.8	Yes
Martínez-Andrade et al.^31^	Primary Care Clinics	Children (aged < 5)	1.5	6	Questionnaire	Screen time	168/138	64.9	Yes
Andrade et al.^33,37^	School	Adolescents	28	-	Questionnaire	Screen time	686/684	79.7	Yes
Leme et al.^26,38^	School	Adolescents	6	-	Questionnaire	Computer/ TV-viewing	142/107	78.2	Yes
Guimarães et al.^27^	School	Adolescents	3	-	Questionnaire	Sedentary activities	49/65	53.1	No
Bandeira et al.^28^	School	Adolescents	3	-	Questionnaire	Screen time	1182	91.8	No
Gutiérrez-Martínez et al.^34^	School	Children	2.5	-	ActGraph Gt3x+	SB ( < 25 counts)	120/68	65	Yes
Rauber et al.^29^	Camp	Children/Adolescents	0.17	3	Questionnaire	Sedentary leisure activities	24	83	No

INT=intervention; CON=control; SB=Sedentary Behaviour; Screen=television, computer and video game time; ITT=intention-to-treat.

**Table 3:** Description of interventions’ components.

	Colín-Ramírez et al.^32^	Bacardi-Gascon et al.^30^	Hardman et al.^25,35,36^	Martínez-Andrade et al.^31^	Andrade et al.^33,37^	Leme et al.^26,38^	Guimarães et al.^27^	Bandeira et al.^28^	Gutiérrez-Martínez et al.^34^	Ruber et al.^29^
Components										
Educational component (meetings/lessons/seminars on health-related subjects)^1^	X	X			X	X	X	X		X
Information (posters, newsletters, guidelines)			X		X	X		X		
PE curriculum modification	X		X					X		
Parent’s involvement	X	X		X		X		X	X	X
Extra PE/PA sessions (before/after school time or during school breaks)						X	X	X	X	X
Exercise breaks in the classroom	X									
Healthy SMS						X			X	
Environmental modification (building facilities or purchasing simple sports equipment)			X		X			X		
PA/sports events on weekends			X							
Strategies target in	PA, SB and diet	PA and diet	PA and diet	PA and diet	SB and PA	PA and diet	PA and diet	PA and screen-time	PA	PA and diet
Professionals responsible for delivering the intervention										
PE teacher/PA professional (or student)		X	X			X	X	X	X	X
Nutritionist (student or professional)		X		X		X	X			X
Other professional	Health Professionals			Health Educator	Researchers				Psychologists	Psychologists and Pedagogues

1.Including lessons taught by another professional other than the school’s Physical Education teacher; PA=physical activity; SB=Sedentary behavior; PE=Physical Education.

Schools promoted environmental modification by building bike racks^25^ and a walking trail^33^ and buying simple sports equipment for Physical Education classes^25^. (Table 3)

All interventions applied strategies to increase PA, and seven also focused on improving diet. Three studies proposed specific strategies to reduce SB: 1) a textbook for teachers and a workbook for adolescents, containing topics related to PA and screen time behavior (i.e. being active for at least 60 min/day and watching television for < 2 hours a day), to be used on classes^33^; 2) recommendations, handed to parents, including decreasing SB-activities time, like TV-viewing, using a computer, or playing videogames^32^; 3) pamphlets on screen time and health for both students and parents^28^. Four studies relied in professionals and students of both Physical Education/Activity and Nutrition for implementing the intervention strategies^26,27,29,30^.

### Results for Sedentary Behavior

Eight interventions reported a positive effect in total SB or recreational screen time reduction^26^. Three effectively reduced the mean time spent in screen based activities to minutes per day (Table 4). One reduced the proportion of adolescents involved in recreational screen time for ≥ 2 hours per day (TV: boys = -8.9%; girls = -7.2%)^28^. The effect size for total SB ranged from -298.9 to -177.1 min/week, and -22.3 to -21.2 min/day (Table 4).

**Table 4:** Interventions’ results

Sedentary time	Effect size	Effect
Martínez-Andrade et al.^31^: no effect on SB.	Mean difference of screen time -1.6 (95%CI: -4.4–1.1)	NE
Andrade et al.^33^: 18 months - the intervention group lower increased total screen time on a weekend day (β = -25.9min/day; p = 0.03) and in the proportion of adolescents exposed to screen time for > 180min/day; 28 months – greater increases in total screen time on a weekday (β = 21.4min/day; p=0.03) were observed among adolescents from the intervention group.	Mean difference of screen time on a weekday 21.2 min/day (SE :13.3)	P
Leme et al.^26^: the intervention group reduced total sedentary activities on weekends (-0.92hrs/day; p = 0.01) compared to the control group.		P
Guimarães et al.^27^: the intervention group reduced the total sedentary time (5652.1; sd = 241.4 to 5641.0; sd = 244.7min/week; p = 0.04) and daily sedentary time (1589.22; sd = 76.4 to 1556.0; sd=73.7min/day; p = 0.01) and presented lower mean compared to control group (6333.7; sd = 177.2min/week; p = 0.04 and 1697.16; sd = 55.3min/day; p = 0.01).	Mean difference of total SB -298.9 min/week (SE: 424.4) Mean difference for screen time -55.5 min/day (SE: 406.8)	P
Gutiérrez-Martínez et al.^34^: the intervention group reduced SB in 11.5 (EE = 8.8; MARA+SMS) and in 15.8 (EE = 10.05; MARA) differently from the control group, which has increased SB in 10.9 (EE = 9.07; p = 0.003) min/day.	Mean difference of total SB -22.3 min/day (SE:17.9)	P
Rauber et al.^29^: participants reduced the time spent in sedentary leisure activities by 177 min/weekdays (p = 0.004) and by 41 min/weekends (p = 0.001).	Mean difference of SB -177.1 min/weekdays -41.1 min/weekends	P
Sitting time		
Bacardi-Gascon et al.^30^: reduced from 9.94 (sd = 2.39) to 9.45 (sd = 1.91) hrs/day	Mean difference of sitting time -0.49 min/day (SE :0.1)	P
TV-viewing		
Colín-Ramírez et al.^32^: had no effect on TV time.	Percentage of children with at least 1 hour a day of TV time -4%	NE
Bacardi-Gascon et al.^30^: reduced from 1.84 (sd = 1.17) to 1.69 (sd = 0.90) hrs/day (p = 0.02)		P
Andrade et al.^33^: 18 months – the intervention group lower increased TV-viewing on a weekday (β = -15.7min/day; p = 0.003) and a weekend day (β = -18.9min/day; p = 0.005); 28 months – Greater increases in TV-viewing (β = 13.1min/day; p = 0.02) were observed among adolescents from the intervention group.	Exposures to total screen time > 2hrs/day Week: OR = 0.94 (95%CI:0.72–1.22) Weekend: OR = 1.05 (95%CI:0.76–1.43)	P
Bandeira et al.^28^: the intervention group reduced the time of TV to less than 2hrs/day (boys: -8.9%; p = 0.005; girls: -7.2%; p = 0.032). Boys in the intervention group had a greater chance to reduce TV use to < 2hrs/day (OR = 2.86; p = 0.037) compared to boys in the control group.	Odds ratio for reducing the use of screens < 2hrs/day Boys: OR = 3.79 (95%CI: 0.5–29.7) Girls: OR = 2.73 (95%CI: 0.5–15.1)	P
Video game/computer		
Colín-Ramírez et al.^32^: 6 months – no effect; 12 months – the intervention group significantly reduced the amount of hours playing video games (23% to 13%; p = 0.001) among children who spent more than 3hrs/day involved in this activity at baseline. This reduction was not observed in the control group (22% to 20%).		P
Hardman et al.^25^: the proportion of adolescents exposured to video game/computer on weekend days for > 2hrs/day was greater in the control group than in the intervention group (29.8% vs. 35.6%; p = 0.004). After adjusting for potential confounding factors, results were not maintained.		NE
Leme et al.^26^: the intervention group reduced computer time (-0.63hrs/day; p = 0.02) compared to the control group.	Mean difference for total screen-time Week: -0.19 (SE: 0.3) Weekend: -0.9 (SE: 0.4)	P
Bandeira et al.^28^: girls in the intervention group reduced the time of computer/video game to less than 2hrs/day (-11.03%; p = 0.0.002). Girls and adolescents aged from 11 to 13 in the intervention group had a greater chance to reduce computer/video game use to < 2hrs/day (girls: OR = 3.34; p < 0.001; 11 to 13 years old: OR = 3.08; p = 0.011) compared to the control group.		P

NE = no effect; P = positive effect – result statistically significant.

Andrade et al. (2015)^33^observed that the intervention group showed smaller increases in screen time compared to the control group for the mean total on a weekend day (intervention: 88.1; control: 112.3 minutes a day) and for the proportion of adolescents with screen time behaviors of > 3 hours a day (intervention: 17.4%; control: 22.7%) after 18 months. Changes were not maintained after 28 months.

Hardman et al. (2014)^25^ observed the benefit of the program *Saúde na Boa* on the proportion of adolescents in the intervention group exposed to videogame/computer on weekend days for > 2 hours compared to the control group (intervention: 29.8%; control: 35.6%), which was not maintained after adjusting for potential confounding factors.

**Figure 2:**
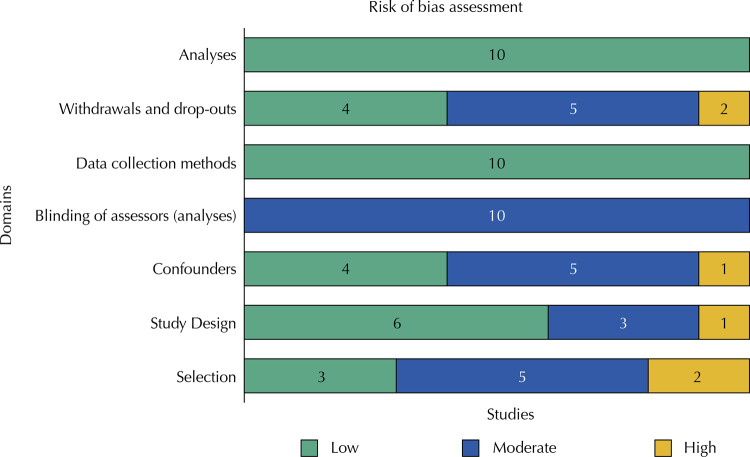
Risk of bias

### Risk of Bias

Most studies included in the syntheses were classified as low risk of bias in the domains: study design (n = 5)^25,26,30,31,33^, data collection methods (n = 10), analyses (n = 10), and the use of intention-to-treat (n = 6)^25,26,30,31,33,34^ approaches. A higher proportion of studies were scored as moderate risk of bias in the domains of selection^25^, blinding of assessors^25^, and withdrawals and drop-outs^26,27,31,33,34^. For the domain of confounders, four studies presented low risk of bias^28,30,31,33^, four presented moderate^26,27,32,34^ and one high risk^25^. Other studies scored high risk of bias for the domains of withdrawals and drop-outs^25,29^, and selection^31^.

## DISCUSSION

Based on the results of ten community-based trials, the descriptive syntheses showed that the most effective community-based interventions were implemented in schools, adopted educational strategies, such as meetings, lessons or seminars on health-related subjects, targeted increasing PA, and were applied by a Physical Education/PA professional.

We found that effective interventions targeted changes in multiple health behaviors, corroborating Grieken et al. (2012)^39^, which reported no difference in the positive effects of interventions of single or multiple health behavior on SB. In this review, the combination of strategies to increase PA, reduce SB, and improve diet were beneficial for SB.

Only a few studies aimed specifically at reducing SB and applied strategies focused on it^25,32,33^. The main strategies were: (1) recommending for parents a more active lifestyle and decreasing SB time spent on television, computer or videogame^32^; and (2) discussing SB and its guidelines in the classroom by a school teacher guided by a didactic material^33^.

Colín-Ramírez et al (2010)^32^ implemented the first strategy, recommending for parents a more active lifestyle, and, after 12 months, the intervention group reduced the daily number of hours playing videogames. The second strategy, discussing SB in the classroom, was still effective after 18 months-follow-up, but not after 28 months. This result is consistent with the systematic review and meta-analysis of Maniccia et al. (2012)^40^of interventions targeting reducing children’s screen time, in which the authors observed larger statistically significant effects during the intervention period than during follow-up^40^.

Screen time was the most prevalent type of SB investigated, even among interventions implemented at schools, where children and adolescents spend a great part of their day sitting and a relatively small amount of time using electronic media for recreational purposes.

Schools are an ideal setting for interventions promoting healthier behaviors and reducing time spent in sedentary activities, as they allow interdisciplinary and multisectoral actions and facilitate parents’ involvement, favoring changes in family’s behavior. Schools also enable beneficial environmental modifications, such as building bike racks and walking trail, and providing sports equipment to be use during the children’s breaks^25,33^.

Previous reviews^20,21^ observed that interventions effectively controlled and/or reduced recreational screen time (the most prevalent type of SB within young people)^3^ among children and adolescents even in low-, middle- and upper-middle-income countries^12^.

In our review, studies evaluated recreational screen time and other types of SB using a questionnaire. Although questionnaires are the most common tool applied to measure SB, they might not be the most accurate for relying solely on participants’ or their parents’ memories to report activities done over a period of time. Yet, objective measurements express a general SB measure, precluding the identification of the contexts in which this behavior has been adopted. A single study within our syntheses applied an objective measurement and not a questionnaire^33^.

Although at-home recreational screen time is above the recommended^10,11^, children and adolescents also spend a lot of time in SB in others settings, especially at school, while attending classess or during the breaks.

Effective interventions included the following educational strategies: school board and teachers meeting, to create a supportive environment for health behaviors; parents education sessions^30^; and textbooks for teachers and workbooks for adolescents on PA and SB, discussed over class.^33^ The most prevalent strategies applied were distributing guidelines and newsletters on nutrition and PA,^26^ and arranging instructional meetings^27^.

Our results showed that seven studies in Latin America adopted family involvement^26,28^, which was effective when combined with educational strategies, information, exercise breaks in the classroom, extra PA sessions and health messages^26,29,32^. Biddle et al. (2014)^41^ review, on interventions to reduce SB in young people, also identified family involvement as an effective strategy. Although some authors observed a more favorable trend in interventions with children younger than six years, we found only one study within this age group^31^ and its intervention was focused on PA and dietary habits, not affecting SB.

Schmidt et al. (2012)^20^ and Wu et al. (2016)^21^ reported that electronic monitor devices, contingent feedback, clinical counseling, and classroom-based health curriculum were effective in reducing screen time among children and adolescents. In both of these reviews, all but one study, conducted in Mexico, were in high-income countries and, as aforementioned, SB determinants and correlates differ according to country’s culture and resources^22,33,42,43^. This finding reinforces the need to test whether intervention strategies to reduce SB in children and adolescents in high-income countries are also relevant in low-, middle- and middle-upper income countries.

In Latin America, most of the effective interventions lasted at least six months,^26,30,32,33^ similar to studies conducted in high-income countries^21^ and in line with the minimum length recommended to promote behavior change^44^.

The main limitation of our review is lack of searches in non-indexed Latin American journals and grey literature, which might have excluded studies that reported no intervention effect. Moreover, our evidence comprises studies from a small number of Latin America countries (n=4).

Our investigation was the first to summarize the effect and characteristics of Latin America interventions to control/reduce SB among children and adolescents. Another strength is the risk of bias assessment. However, as the high risk of bias in dropout and selection rates could play a role in the non-effect of some interventions^23,29^, results should be interpreted with caution.

Our findings indicate gaps and a need for further studies that (i) define SB as a primary objective and implement strategies to reduce it; (ii) target sedentary activities and settings other than at-home screen time and time spent sitting in the classroom; (ii) use objective tools together with questionnaires to measure SB, informing a more reliable SB time and which settings and types of sedentary activity are more common among young people; (iv) conduct interventions in Latin America countries other than Brazil, Mexico, Ecuador, and Colombia.

Most Latin America interventions did not define SB as a primary objective or applied strategies specific to it. Yet, they effectively reduced SB, mainly recreational screen time, among children and adolescents. Effective interventions were conducted at school and often applied educational and informative strategies, as meetings, seminars, workshops, and distribution of guidelines and newsletters.

These results are important for public managers to plan actions to reduce SB among children and adolescents considering contexts and activities other than at-home leisure time, as young people are also sedentary at school and transportation.
